# Juniper camphor: An emerging sesquiterpene compound with promising chemistry, biological potential, and future perspectives

**DOI:** 10.1007/s44446-026-00082-2

**Published:** 2026-04-30

**Authors:** Salma Ebrahim Mohammed, Abdullah Haikal

**Affiliations:** 1 Faculty of Pharmacy, Mansoura University, Mansoura, 35516 Egypt; 2 Department of Pharmacognosy, Faculty of Pharmacy, Mansoura University, Mansoura, 35516 Egypt

**Keywords:** Juniper camphor, Essential oil, Antimicrobial, Antioxidant, Anti-inflammatory, Phytotherapy

## Abstract

Juniper camphor, a sesquiterpene alcohol with selinane-type skeleton, which is a subset of the eudesmane family generated predominantly from several *Juniperus* species, is an important phytochemical having medicinal benefits. Juniper camphor, also called eudesm-7(11)-en-4-ol, is a sesquiterpene having the chemical formula C_15_H_26_O. It is largely made from the essential oils of numerous juniper species, including *Juniperus communis* and *Juniperus californica*. Methods of extraction, botanical distribution, and environmental factors can influence the yield of juniper camphor extracted from natural populations**.** Juniper camphor has gained attention for its diverse applications in traditional medicine, aromatherapy, and the fragrance and flavor industries. Recent studies highlight its significant antimicrobial, anticancer, anti-inflammatory, and antioxidant properties in addition to its allelopathic activity. They are significant prospects for further research in the field of natural health therapies because of their many benefits.

## Introduction

Essential oils are complex, naturally occurring hydrophobic liquids. They are characterized by their aromatic properties and their ability to evaporate at room temperature (Abd Rashed et al. 2021). Usually extracted as secondary metabolites from fragrant plants, these oils consist of a wide variety of organic compounds, mainly terpenes and their oxygenated form, classified by the number of isoprene units into monoterpenes and sesquiterpenes, which play a role in their therapeutic characteristics (Moullamri et al. 2025; Trepa et al. 2024). Additionally, their structural diversity and relatively high lipophilicity enable them to interact with biological membranes and protein targets, making them promising candidates for drug discovery and agrochemical development (Korać and Khambholja 2011; Park et al. 2009).

Terpenes are widely found natural compounds recognized for their remarkable structural variety and significance in medicinal applications, fragrance components, and as alternative biofuels (Câmara et al. 2024; Peralta-Yahya et al. 2012; Reuter et al. 2025). Terpenoids are generated from widely available isoprenyl diphosphate precursors, which are synthesized through either the mevalonate (MEV) pathway or the 1-deoxyxylulose-5-phosphate (DXP) pathway (Kirby and Keasling 2009; Pichersky and Raguso 2018; Trapp and Croteau 2001).

**Fig. 1:**
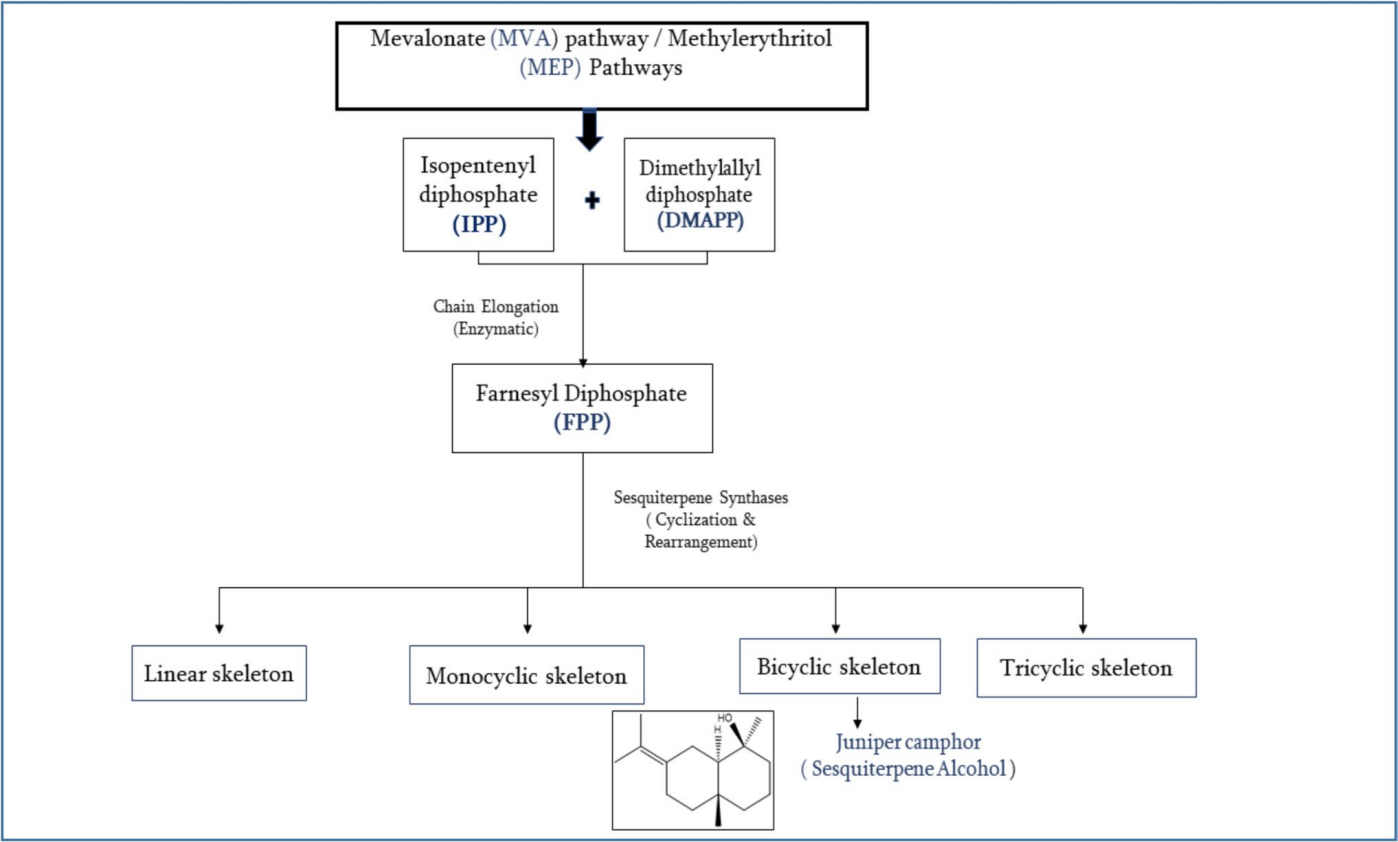
The formation of sesquiterpene skeletons from farnesyl pyrophosphate (FPP) occurs through enzymatic cyclization

Sesquiterpenes are a diverse class of terpenoid compounds composed of three isoprene units, resulting in the molecular formula C_15_H_24_ for their hydrocarbon forms, which occur naturally in insects and higher plants (Bakkali et al. 2008). They are biosynthesized via the mevalonate (MVA) or methylerythritol phosphate (MEP) pathways, starting from the universal five carbon precursors: isopentenyl diphosphate (IPP) and dimethylallyl diphosphate (DMAPP). These precursors undergo sequential condensation to form farnesyl diphosphate (FPP), the central C_15_ intermediate in sesquiterpene biosynthesis. FPP is then cyclized or rearranged by sesquiterpene synthases to yield a vast array of linear, monocyclic, bicyclic, and tricyclic skeletons (Asadollahi et al. 2010; Dickschat 2016), and they may also contain various functional groups that boost their biological functions (Fig. 1).

Motl et al. identified a sesquiterpene alcohol derived from juniper berry oil and elucidated its molecular structure. This marked the first formal recognition and naming of "juniper camphor" which belongs structurally to the selinane type skeleton, which is part of the eudesmane family (Motl et al. 1958).

Beyond its chemical and structural interest, juniper camphor holds significant applications in pharmaceuticals, cosmetics, and food flavoring due to their antimicrobial, anti-inflammatory, diuretic, antidiabetic, and antioxidant properties, as well as their anticarcinogenic, hepatoprotective, neuronal, and renal effects, suggesting its potential as a bioactive natural compound. Moreover, its molecular structure provides a promising scaffold for the development of novel therapeutic agents. The growing interest in plant derived compounds in drug discovery further highlights the importance of systematically evaluating juniper camphor from a pharmacological perspective (Arizmendi et al. 2022; Fierascu et al. 2018; Kuete 2013; Laouar et al. 2017; Popescu et al. 2023).

Despite the growing interest in juniper camphor and its reported biological activities, several gaps remain in the current literature. Most available studies focus primarily on preliminary pharmacological screening, while comprehensive mechanistic investigations are still limited. In addition, clinical evidence supporting its therapeutic applications remains scarce, and safety profiles are not yet fully characterized. Furthermore, existing data are often fragmented across different experimental models, making it difficult to draw consistent conclusions. Therefore, a comprehensive and critical review of the available evidence is necessary to clarify current knowledge, identify research gaps, and propose future research directions.

## Botanical sources and distribution of juniper camphor

Juniper camphor, primarily derived from various species of the *Juniperus* genus, is a significant phytochemical with notable therapeutic properties. Juniper camphor is a sesquiterpene compound with the molecular formula C_15_H_26_O. It is primarily derived from the essential oils of various juniper species, such as *Juniperus communis* and *Juniperus californica*. This compound is characterized by its complex bicyclic structure and its distinctive woody camphoraceous odor. Juniper camphor has gained attention for its diverse applications in traditional medicine, aromatherapy, and the fragrance and flavor industries (Hymete et al. 2007; Motl et al. 1958). Beyond the *Juniperus* genus, juniper camphor has been identified in several other plant families. For instance, it accounts for 14.3% of the root essential oil of *Echinops kebericho*, traditionally used in Ethiopian ethnomedicine for its antimicrobial and anti-inflammatory properties (Hymete et al. 2007). High proportions have also been recorded in *Pulicaria somalensis* (24.7%), whose sesquiterpene rich oil showed potent antioxidant and allelopathic effects (Assaeed et al. 2020).

**Fig. 2:**
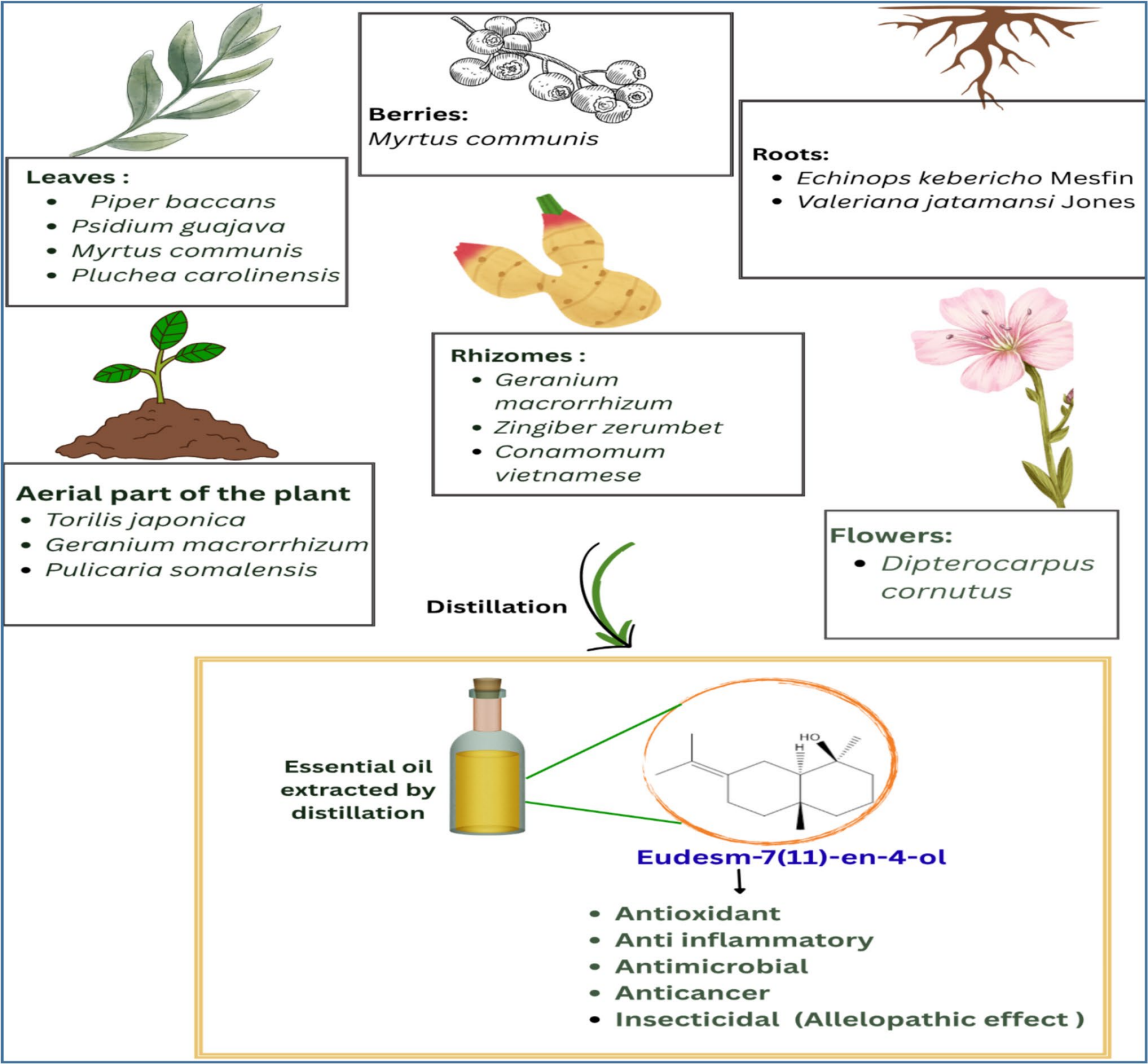
An overview illustrating the various botanical sources utilized for the extraction of juniper camphor

The genus *Juniperus* is a key member of the Cupressaceae family, with over 50 species and 36 varieties recognized globally (Adams and Pandey 2003). *Juniperus* thrives in temperate regions of the northern hemisphere, including Asia, Europe, Africa, and North America (Dahmane et al. 2015). The distribution of juniper across the Eurasian supercontinent spans from the eastern Mediterranean and the Balkans through Turkey, reaching the mountains of Iran, Afghanistan, and extending into Pakistan (Al-Refahi et al. 2002). Previous studies indicate that juniper populations in the mountain forests of Iran's central plateau are distributed at elevations ranging from 1700 to 2500 m (Ravanbakhsh et al. 2013). Juniper camphor can also be extracted from other plants and different plant parts, as shown in Fig. 2.

## Juniper camphor chemistry

**Fig. 3:**
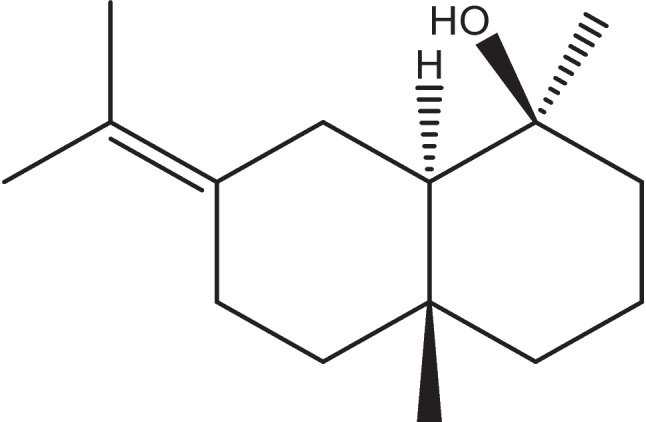
Chemical structure of juniper camphor (eudesm-7(11)-en-4-ol)

A sesquiterpene alcohol (Fig. 3) with the molecular formula C_15_H_26_O is recognized for its intricate bicyclic structure. It is classified structurally as belonging to the selinane-type skeleton, which is a subset of the eudesmane family. Eudesm-7(11)-en-4-ol, commonly known as juniper camphor, occurs in several stereoisomeric forms that have been identified and characterized through comprehensive spectroscopic methods, including NMR, mass spectrometry, FTIR, and Kovats indices. These stereoisomers display structural variations that may influence biological activity and play a role in distinguishing plant species and their chemotaxonomic classification (Kesselmans et al. 1992).

## Methods of extraction

Juniper camphor is primarily extracted from various aromatic plants, particularly species of *Juniperus*, as part of the essential oil fraction. The extraction methods used can significantly affect both the yield and composition of the oil. Common techniques include steam distillation, hydrodistillation, solvent extraction, and solid phase microextraction (Govender 2010). One of the extraction procedures is described as a fresh plant was chopped into small fragments and then underwent hydro-distillation for 3 h with Clevenger-type apparatus to extract the oil, which was collected and dehydrated with anhydrous sodium sulfate. The oil was then stored in a sealed vial at low temperatures for GC/MS analysis and biological studies (Haikal et al. 2024).

## Chemical variation and environmental influence on the yield of essential oil

Environmental factors play a crucial role in selecting specialized genotypes or chemotypes that are better adapted to their local conditions. This leads to the development of various chemotypes in response to the complex gradients of climate and soil (Thompson et al. 2003). Additionally, these factors can influence the yield of juniper camphor extracted from natural populations of *Salvia leriifolia* Benth. The major components include oxygenated sesquiterpenes, with juniper camphor concentrations varying between 12.0% and 39.9%. *Salvia leriifolia* populations originating from Semnan province (Shahroud, Iran) are characterized by juniper camphor levels of 21.9% to 28.7%. This study identified two distinct groups of populations based on juniper camphor concentrations. The South Khorasan group exhibited lower levels at 12.0%, while the Razavi Khorasan group (including Mashhad, Najmabad, Soltanabad, and Nakhbar) had significantly higher levels ranging from 32.1% to 39.9%. The essential oil derived from *S. leriifolia* demonstrated high polymorphism, which may be influenced by environmental conditions and genetic factors (Yousefi et al. 2012). Variations in geographic temperature and rainfall may explain patterns in the yield and composition of essential oils. In addition, the developmental stage of plant organs can affect the production and concentration of essential oils; as a result, the yields and compositions of these oils vary with the seasons. The essential oil derived from *Cinnamomum camphora* showed that juniper camphor was present in small amounts. Additionally, correspondence analysis indicated that environmental variables greatly affect both the oil yield and the essential oil composition of *C. camphora*; the highest proportion of juniper camphor was expressed in JX-AF −14 chemotype as 0.78% (Zhang et al. 2023).

## Biological activities

**Fig. 4:**
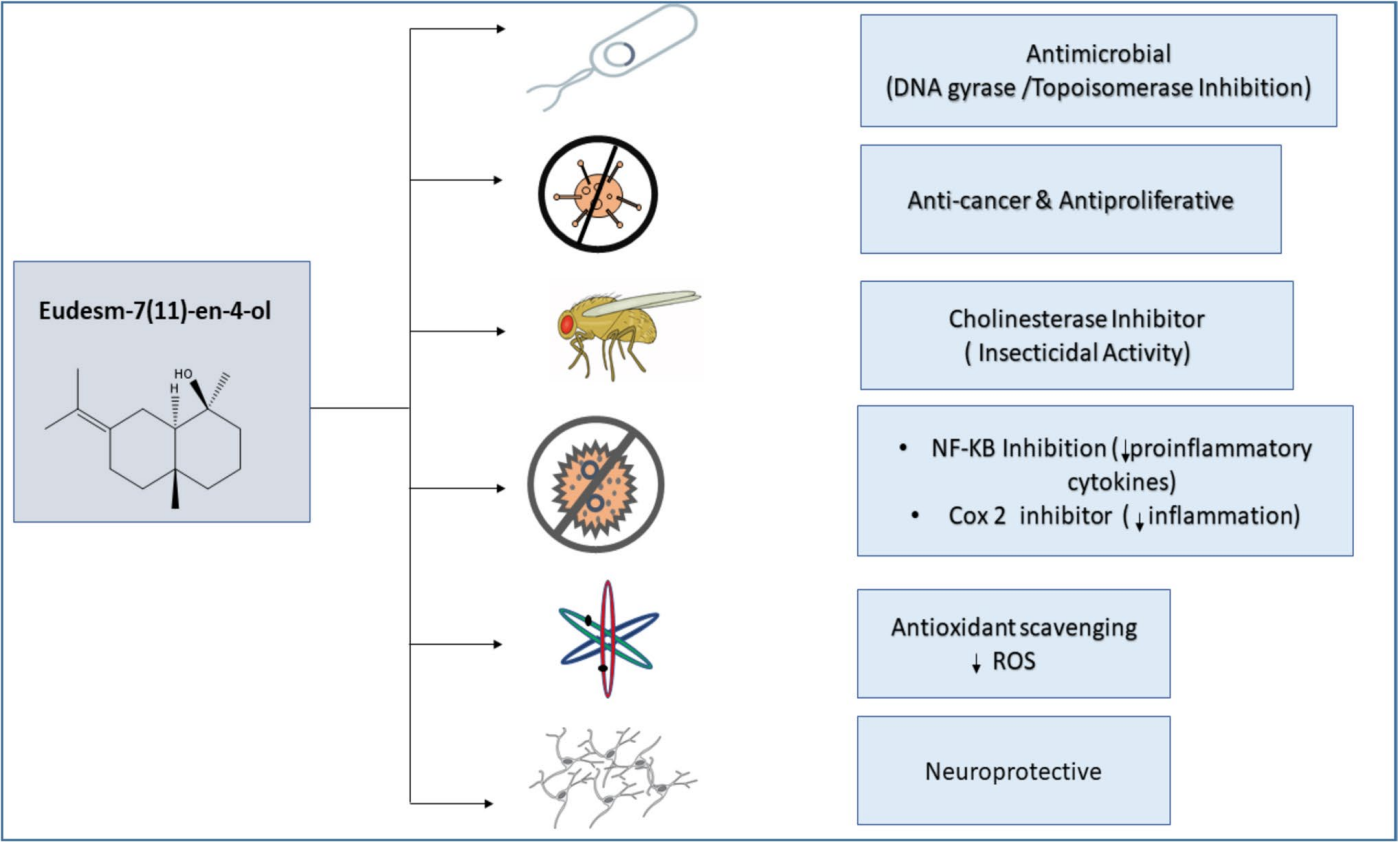
Schematic representation of the reported biological potential of juniper camphor

Oxygenated sesquiterpenes like juniper camphor present in diverse essential oils exhibited a broad spectrum of therapeutic effects (Fig. 4). These substances showed considerable antioxidant, antimicrobial, anti-inflammatory, and anticancer properties. Their diverse advantages make them important candidates for additional investigation in the realm of natural health remedies (Koga et al. 2016; Silva et al. 2018; Sitarek et al. 2017). It is important to emphasize that the majority of the reported biological activities were evaluated using the whole essential oil rather than the isolated juniper camphor. Therefore, direct attribution of the observed effects specifically to juniper camphor remains tentative and requires further validation through studies using the pure compound.

### Antimicrobial activity

Several studies indicate that juniper camphor contributes to the antimicrobial activity of essential oils, showing both antibacterial and antifungal effects (Table 1). In *Tanacetum parthenium* essential oil, where it constituted 6.23% of the composition, the oil showed significant inhibitory effects against *Staphylococcus subtilis*, *Enterobacter aerogenes*, and *Escherichia coli*, with minimum inhibitory concentrations (MIC) as low as 4 μL/mL for certain strains (Mohsenzadeh et al. 2011). The essential oil from *Asarum geophilum* contains juniper camphor at a concentration of 13.41%, while essential oil from *A. splendens* contains 14.21%. *A. geophilum* showed significant inhibitory effects against *Escherichia coli*, *Pseudomonas aeruginosa*, *Fusarium oxysporum*, and *Saccharomyces cerevisiae*, with minimum inhibitory concentration (MIC) values of 200 µg/mL for all. The essential oil from *A. splendens* demonstrated significant inhibitory activity against *Escherichia coli, Saccharomyces cerevisiae,* and* Fusarium oxysporum*, showing MIC values of 200 µg/mL (Minh et al. 2023).

**Table 1:** Juniper camphor containing plants with antimicrobial activity

Plant	Juniper camphor percentage by GC/MS	Experimental Details	Reference
*Lepechinia mutica* (Benth.) Epling	Juniper camphor (13.02%)	*In-vitro* antifungal activity: *Pyricularia oryzae* (LM120) MIC 0.0125–0.0250 mg/ml, MFC > 0.1000 mg/ml; *Microsporum canis* (CBS 136538) MIC 0.0250–0.0500 mg/ml, MFC 0.0500–0.1000 mg/ml	(Ramírez et al. 2018)
Volatile constituents of the roots of *Echinops kebericho* Mesfin	The main constituent found in the hydrodistilled essential oil was (Juniper camphor 14.3%)	MIC = 78.125 μg/ml for methicillin-resistant *Staphylococcus aureus* (MRSA, NCTC 12493)	(Hymete et al. 2007) (Deyno et al. 2021)
The essential oil from the aerial parts of *Torilis japonica*	Juniper camphor (10.42%)	*In-vitro* antibacterial activity (agar disk diffusion and broth microdilution): *B. subtilis* MIC 0.16 mg/ml	(Chen et al. 2013)
Essential oil of *Chrysanthemum morifolium* capitulum	The main chemical constituent was juniper camphor	MIC = 125 µg/ml against *Bacillus Subtilis*, *Streptococcus Pyogenes,* and *Streptococcus Agalactiae*	(Guo et al. 2008) (Youssef et al. 2020)
Essential oil from *Tanacetum parthenium*	Juniper camphor (6.23%)	MIC = 4 µL/mL against *Staphylococcus subtilis* and 38 µL/mL against *Enterobacter aerogenes*	(Mohsenzadeh et al. 2011)
Essential oil from *Valeriana jatamansi* Jones roots and rhizomes	Juniper camphor (minor)	Effective against *Staphylococcus aureus* and *Escherichia coli*, with MICs of 1.5 μg/mL and 0.8 μg/mL during sun drying	(Thakur et al. 2025)
Essential oils of four *Asarum* species growing in Vietnam	The *A. geophilum* contains juniper camphor (13.41%) while *A. splendens* contains juniper camphor (14.21%)	*A. geophilum* inhibited *Escherichia coli, Pseudomonas aeruginosa, Fusarium oxysporum,* and *Saccharomyces cerevisiae* with MIC values of 200 µg/mL*. A. splendens* showed similar activity against* Escherichia coli, Saccharomyces cerevisiae,* and* Fusarium oxysporum,* also at 200 µg/mL	(Minh et al. 2023)

The Ecuadorian plant species *Lepechinia mutica* (Benth) Epling has demonstrated significant antifungal effects against *Pyricularia oryzae*, the pathogen responsible for blast disease. This activity is attributed to the secondary metabolite carnosol, with juniper camphor identified as the major component of the essential oil, accounting for 13.02%. The minimum inhibitory concentration (MIC) of the essential oil against *Pyricularia oryzae* ranges from 0.0125 mg/ml to 0.0250 mg/ml. Furthermore, *Lepechinia mutica* also exhibits activity against *Microsporum canis*, with an MIC ranging between 0.025 mg/ml and 0.050 mg/ml (Ramírez et al. 2018). The essential oil extracted from the aerial parts of *Torilis japonica* showed a concentration of juniper camphor at 10.42%. This oil exhibited antibacterial properties against food spoilage organisms, successfully inhibiting *S. typhimurium*, *B. subtilis*, and *L. monocytogenes*. Among these, *B. subtilis* was the most susceptible strain, with MIC of 0.16 mg/ml (Chen et al. 2013). Essential oil extracted from the roots and rhizomes of *Valeriana jatamansi* Jones contains a small amount of juniper camphor. The samples dried using sun and shade techniques yielded the highest levels of juniper camphor, measuring 0.84% and 0.86%, respectively. Additionally, essential oils derived from the roots and rhizomes showed effectiveness against *Staphylococcus aureus* and *Escherichia coli*, with minimum inhibitory concentrations (MICs) of 1.5 μg/mL and 0.8 μg/mL, respectively, at sun drying conditions. (Thakur et al. 2025). Screening of the essential oil extracted from the roots of *Echinops kebericho* Mesfin revealed that the primary component identified in the hydrodistilled essential oil was juniper camphor, which constituted 14.3%. The roots demonstrated prominent anthelmintic effects, boasting an LD_50_ of 0.057 mg/ml, which was more effective than niclosamide (Hymete and Kidane 1991; Hymete et al. 2007). The essential oil of *Echinops kebericho* demonstrated antimicrobial and antioxidant properties, and furthermore, *E. kebericho* showed potent antileishmanial activity that surpassed that of amphotericin B, with notable cytotoxicity in two strains of *Leishmania* (*L. aethiopica* and *L. donovani*). This activity was observed against both the promastigote (MIC 0.0097–0.1565 ml/ml) and axenic amastigote forms (EC_50_ 0.24–42.00 nl/ml) of the *Leishmania* species (*L. aethiopica* and *L. donovani*) (Deyno et al. 2021; Tariku et al. 2011). The essential oil derived from *Pluchea carolinensis* has been analyzed, revealing one hundred thirteen different compounds, with juniper camphor being the most prevalent at 37.6% (Pino et al. 2005). *Pluchea carolinensis* oil exhibits significant antileishmanial properties against *Leishmania amazonensis. In-vitro* studies demonstrated that* P. carolinensis* essential oil inhibited the growth of promastigotes (IC_50_ = 24.7 ± 7.1 μg/mL) and amastigotes (IC_50_ = 6.2 ± 0.1 μg/mL). In a study utilizing BALB/c mice to model experimental cutaneous leishmaniasis, administering five doses of essential oil at 30 mg/kg via the intralesional route resulted in reduced lesion size and parasite load (p < 0.05) compared to mice that were treated with Glucantime and those that received no treatment. To conclude, both *in-vitro* and* in-vivo* findings indicated the promising potential of essential oil from *P. carolinensis* as a prospective alternative treatment for leishmaniasis (García et al. 2017). Moreover, it demonstrated positive antibacterial effects against *Staphylococcus aureus* and *Bacillus subtilis* (Córdova et al. 2006). Furthermore, it exhibited notable antioxidant qualities, in addition to anti-inflammatory, hepatoprotective, cytotoxic, anti-ulcer, antipyretic, antiviral, antinociceptive, and anti-amoebic actions (Ahemd and Kamel 2013; García et al. 2017; Hervet et al. 2006; Hussain et al. 2013; Perera et al. 2014). Research on eudesmane type sesquiterpenes with similar structures indicated that their antimicrobial properties might include the inhibition of bacterial enzymes like DNA gyrase or type II topoisomerase, which leads to interference in DNA replication and cellular division (Mohamed et al. 2023).

### Antioxidant and anti-inflammatory properties

Reactive oxygen species (ROS) are generated by the partial decrease of oxygen during aerobic metabolism (Lushchak 2014). Hydroxyl radicals (OH), hydrogen peroxide (H_2_O_2_), and superoxide anion are a few instances of ROS. Under normal conditions or low levels of oxidative stress, the body's built-in antioxidant defense system helps cells fight off potential harm by using the right enzymes, like glutathione (GSH) reductase, GSH peroxidase, superoxide dismutase (SOD), and catalase, to detoxify ROS. However, oxidative stress may be triggered by an imbalance in the antioxidant defense system or an excess of free radicals that surpasses the cell's ability for detoxification (Rahman 2007). Oxidative stress has been shown to harm biomolecules, including proteins, DNA, and lipids, leading to cellular damage (Uttara et al. 2009).

Sesquiterpene alcohols consistently target the innate immune signaling pathway, evidenced by the downregulation of NF-κB activity in patchouli alcohol and farnesol, and are associated with diminished systemic and tissue cytokine levels in infected and toxicant models. They also suppress TLR4 expression and signaling, which contributes to downstream NF-κB activity, leading to cytokine suppression (Alruhaimi et al. 2024; Jiang et al. 2025; Lv et al. 2025).

Studies carried out using *in-silico* methods suggest that juniper camphor and comparable compounds might interact with proteins linked to inflammation and oxidative stress, underscoring their potential antioxidant properties at the molecular scale. The study identified juniper camphor as a moderate inhibitor of cyclooxygenase-2 (COX-2), with a binding free energy comparable to the pharmaceutical reference meloxicam (Xiao et al. 2024). This suggests potential for anti-inflammatory drug development, pending further *in-vitro* and *in-vivo* validation. Essential oil of *Solanum lyratum* Thunb was evaluated for COX-2 inhibition through molecular docking and dynamic simulations, with juniper camphor showing a percentage of 1.64% (Xiao et al. 2024).

Juniper camphor containing essential oils has been linked to strong antioxidant capacities (Table 2). *Pulicaria somalensis* essential oil, which contained 24.7% juniper camphor, exhibited antioxidant activity comparable to ascorbic acid in DPPH and ABTS radical scavenging assays with IC_50_ of 81.2 mg/ml and 64.4 mg/ml, respectively (Assaeed et al. 2020). Similarly, compared to* A. splendens* essential oil (SC_50_ value of over 100 µg/mL)*, Asarum geophilum* essential oil*,* which contains 13.41% juniper camphor, showed significant antioxidant capability with a SC_50_ value of 28.57 µg/mL (Minh et al. 2023). Phytochemical comparison of *Salvia mirzayanii* Rech. & Esfand in different ecological conditions, the results of oil analysis indicated that the maximum concentration of juniper camphor was observed in ecotype 3 and ecotype 1 (23.56 and 14.63%, respectively), analyzed by GC-FID and GC/MS. According to these results and the comparison of means, the lowest IC_50_ (best antioxidant activity) was related to ecotype 3. It showed a moderate antioxidant activity in comparison with *α*-tocopherol (Vitamin E) as a standard, has many pharmacological effects, including antioxidant, anti-cholinesterase, antimicrobial, anticancer, anti-inflammatory, and enhancing cognition and memory (Ghasemi et al. 2023). The acetone extract from the rhizome of *Zingiber zerumbet* showed the presence of juniper camphor at a concentration of 2.87%. Research findings highlighted that *Z. zerumbet* is a significant source of sesquiterpenes and oxygenated sesquiterpenes, which are suitable for large-scale industrial applications in the production of pharmaceuticals, fragrances, and flavoring agents (Dash et al. 2020). The essential oil of *Eriocephalus africanus,* containing 14.17% sesquiterpene juniper camphor, was shown for the first time to have hepatoprotective properties against Con A-induced autoimmune hepatitis (AIH) in mice. This effect was achieved through its antioxidant, anti-inflammatory, and antinecrotic characteristics. These advantageous effects are associated with its capacity to selectively reduce immune cell infiltration in the liver, restore hepatic redox balance, and simultaneously inhibit the TNF-α/NF-κB and IFN-γ/STAT1 signaling pathways (Haikal et al. 2024). The essential oils extracted from various Iranian populations of *Rhabdosciadium aucheri* Boiss showed variation across seven natural populations, with juniper camphor demonstrating antioxidant activity ranging from 3.5% to 20.8% (Kazemeini et al. 2022). The essential oil extracted from the aerial parts of *Geranium macrorrhizum* L. in various regions of Bulgaria has shown the presence of sesquiterpenes such as juniper camphor in acetate form in certain samples. The rhizome of *Geranium macrorrhizum* has demonstrated a lower antioxidant capacity compared to the aerial parts (Tzanova et al. 2024). The essential oil extracted from *Ephedra sinica* in Northeastern China contains juniper camphor in concentrations ranging from 0.41 to 6.13. *E. sinica* has been utilized to address ailments such as the common cold, asthma, bronchitis, and arthritis, and it has also functioned as a CNS stimulant and mood enhancer in dietary supplements in Western cultures (Gurley et al. 1998; Wang et al. 2006).

**Table 2:** Juniper camphor containing plants with antioxidant activity

Plant	Method of identification or isolation	Juniper camphor percentage	Experimental Details	Reference
*Pulicaria somalensis* aerial parts	GC/MS	Juniper camphor (24.7%)	IC_50_ (µg/mL) is 81.2 and 64.4 in DPPH and ABTS, respectively	(Assaeed et al. 2020)
*Zingiber zerumbet* rhizome	GC/MS	Juniper camphor (2.87%) as a minor constituent	Using the DPPH assay, an IC_50_ of 17416.04 ± 3274.95 μg/mL was obtained, while the ABTS assay showed an IC_50_ of 2884.67 ± 232.71 μg/mL	(Dash et al. 2020) (Tian et al. 2020)
*Echinops kebericho* Mesfin roots	GC/MS	The primary component identified in the hydrodistilled essential oil was juniper camphor, which accounted for 14.3% of the total composition	The IC_50_ value of the crude extract was found to be 5.89 ± 1.02 μg/ml, determined using the DPPH assay	(Hymete et al. 2007) (Yimer et al. 2021)
*Solanum lyratum* Thunb	GC/MS	Juniper camphor: 1.64%	In the *in-silico* study, the binding free energy of juniper camphor was observed to be 6.35 kcal/mol	(Xiao et al. 2024)
*Eriocephalus africanus*	GC/MS	Sesquiterpene juniper camphor (14.17%) (Major form)	Essential oil showed *in-vivo* hepatoprotective potential against Con A-induced hepatitis in mice collectively through selective anti-oxidant, anti-inflammatory, and anti-necrotic effects	(Haikal et al. 2024)
*Rhabdosciadium aucheri* Boiss	GC/MS	Juniper camphor (3.5–20.8%)	The methanolic extract showed a higher antioxidant activity, with an IC_50_ value of 0.19 mg/ml, as determined by the DPPH assay	(Kazemeini et al. 2022) (Azimi et al. 2021)
Thyme species (*T. talijevii*, *T. paucifolius*, and *T. punctulosus*)	Trace DSQ chromatograph -mass spectrometer (Thermo)	The essential oils exhibited a substantial presence of bicyclic sesquiterpenes, including juniper camphor	In *Thymus vulgaris* L, the EC_50_ value is 69.39 ± 3.01 μg/ml, by DPPH assay	(Alekseeva and Gruzdev 2012; Mokhtari et al. 2023)
*Pluchea carolinensis*	GC (FID)	One hundred thirteen compounds of *P. carolinensis* oil were identified, with juniper camphor (37.6%) as the major component	The highest DPPH antioxidant potential was frequently observed in fractions obtained from *n*-butyl alcohol	(Pino et al. 2005) (Perera et al., 2014)
*Chenopodium botrys*	GC-FID and GC/MS analysis	Juniper camphor (8.94%) was found as the main compounds of Konia sample	(0.87 mg GALAEs/g oil and 0.82 mg KAEs/g oil)	(Ozer et al. 2017)
The aerial parts of *Salvia mirzayanii* Rech. & Esfand	GC-FID and GC/MS	The results of oil analysis indicated that the maximum concentration of juniper camphor was observed in ecotype 3 and ecotype 1 (23.56 and 14.63%)	The lowest IC_50_ (best DPPH antioxidant activity) was related to ecotype 3 with 1281.48 μg/mL	(Ghasemi et al. 2023)
*Ephedra sinica*	GC/MS	Juniper camphor (0.41–6.13%)	*In-vivo* assay using Sprague–Dawley rats showed strong antioxidant effects, leading to reduced levels of hepatic malondialdehyde (MDA) and other oxidative stress markers such as hydrogen peroxide (H_2_O_2_) and nitric oxide (NO). Additionally, the activities of hepatic superoxide dismutase (SOD) and catalase (CAT) were further increased	(Wang et al. 2006) (Seif et al. 2021)
Essential oils of four *Asarum* species growing in Vietnam	GC/MS	The *A. geophilum* contains juniper camphor (13.41%) while *A. splendens* contains juniper camphor (14.21%)	*Asarum geophilum* Essential oil demonstrated significant antioxidant capability with an SC_50_ value of 28.57 µg/mL using DPPH assay	(Minh et al. 2023)
*Geranium macrorrhizum* L	GC/MS	Juniper camphor in acetate form was detected in essential oils from the aerial parts	DPPH assay showed IC_50_ 0.192,0.196.0.217 and 0.208 mg/ml to the aerial parts	(Tzanova et al. 2024)
*Valeriana jatamansi* Jones roots and rhizomes	GC/MS and gas chromatography-flame ionization detection	Juniper camphor (minor)	DPPH assay showed IC_50_ 269.84 μg/mL in shade drying	(Thakur et al. 2025)

Alongside the antimicrobial capabilities discussed earlier, the antioxidant properties of oils from *V. jatamansi* roots and rhizomes were assessed using the DPPH assay and expressed as IC_50_ values. The findings revealed that the oils of *V. jatamansi* roots/rhizomes exhibited significant antioxidant activity, and the drying conditions had a notable influence (*p* ≤ 0.05) on their antioxidant capacity. Ascorbic acid was utilized as a standard reference antioxidant (IC_50_ 56.89 μg/mL). When compared to the positive control, the antioxidant effectiveness of the *V. jatamansi* roots/rhizome oils was relatively modest. Nevertheless, among the various oil samples, the shade-dried roots and rhizomes showcased the highest antioxidant capacity (IC_50_ 269.84 μg/mL) (Thakur et al. 2025).

In 2017, this study focused on analyzing the chemical components of the essential oil obtained from *Chenopodium botrys* L., which was gathered from three different regions in Turkey. Additionally, the antioxidant properties of the oil samples were assessed using several assays, including free radical scavenging, phosphomolybdenum, ferrous ion chelation, and reducing power tests. It was also investigated their impact on the inhibition of acetylcholinesterase (AChE), butyrylcholinesterase (BChE), and tyrosinase. The results from GC-FID and GC–MS analyses showed that twenty-seven, twenty-four, and sixteen compounds were identified, accounting for 94.45%, 96.96%, and 94.41% of the oils, respectively. The main compound in the Konya sample was juniper camphor, constituting 8.94% of the oil. In the assays for AChE and tyrosinase inhibition, the essential oil of *C. botrys* sourced from Konya exhibited the highest activity (0.87 mg GALAEs (galanthamine equivalents)/g oil and 0.82 mg KAEs (kojic acid equivalents)/g oil) (Ozer et al. 2017).

*Rhetinolepis lonadioides* (Coss.) is a medicinal plant from the Asteraceae family, located in the Bechar region of southwest Algeria, and it possesses a variety of biological properties and medicinal benefits. This research evaluates the potential use of its essential oils as natural agents with antioxidant effects. The essential oil derived from the aerial parts of *R. lonadioides* was analyzed for its chemical composition using chromatographic and spectral techniques, specifically GC-FID and GC–MS, revealing that juniper camphor was present in a minor amount at 0.47%. The study found that the oil from *R. lonadioides* demonstrated greater antioxidant activity *in-vitro*, as indicated by its DPPH free radical scavenging ability, with an IC_50_ value of 2.13 μl/ml (Mansour et al. 2024).

This research outlines the composition of essential oils from three *Litsea* species: *Litsea costalis, Litsea machilifolia,* and *Litsea globularia*, which were gathered from Malaysia and analyzed using GC-FID and GC–MS. A minor quantity of juniper camphor was found exclusively in the essential oil of *L. costalis*, at a percentage of 1.0 ± 0.1 (mean ± SD) (Azhar et al. 2022).

### Insecticidal and allelopathic effects

Juniper camphor also contributes to insecticidal properties (Table 3). In *Psidium guajava* leaf essential oil, juniper camphor (2–2.3%) enhanced toxicity against *Sitophilus zeamais* (maize weevil) in contact assays, with higher activity observed in oils extracted in the afternoon (Usman et al. 2025). The essential oil of *Piper baccans* contains juniper camphor acetate at a concentration of 30.71% and demonstrated significant larvicidal efficacy against *Aedes aegypti*, as shown by its impressive LC_50_ and LC_90_ values. The essential oil is known to inhibit acetylcholinesterase (AChE) and exhibited genotoxic effects on larvae. Importantly, at low concentrations, it was non-toxic to larval predators like *Diplonychus indicus* and *Anisops bouvieri*, making it a viable option for managing insect vectors (Souza et al. 2024). The essential oil extracted from *Laggera pterodonta*, containing 5.51% juniper camphor, exhibited insecticidal activity against adult *Lasioderma serricorne* and *Liposcelis bostrychophila* (Guo et al. 2017). In *Pulicaria somalensis*, oil containing juniper camphor exhibited allelopathic properties, suppressing the germination and growth of various weeds. The essential oil displayed considerable allelopathic effects against *Dactyloctenium aegyptium* (crowfoot grass) and *Bidens pilosa* (hairy beggarticks). The IC_50_ value for the germination of *D. aegyptium* was double that of *B. pilosa*. Regarding root growth, the IC_50_ values for both *B. pilosa* and* D. aegyptium* were measured at 0.6 mg/mL, whereas for shoot growth, the values were 1.0 mg/mL for *B. pilosa* and 0.7 mg/mL for *D. aegyptium*. This difference in effectiveness could be linked to the genetic traits of the weeds, indicating possible uses in sustainable pest and weed management (Assaeed et al. 2020).

**Table 3:** Juniper camphor containing plants with insecticidal potential

Plant	Juniper camphor percentage by GC/MS	Experimental Details	Reference
*Psidium guajava* leaves	Juniper camphor (minor): 2.3% in the morning and 2% in the afternoon	LT_50_ 121.09 h in 7 a.m and 59.23 h at 1 pm against *S. zeamais*	(Usman et al. 2025)
*Laggera pterodonta*	Juniper camphor (5.51%)	LD_50_ 32.97 mg/adult aginst *Lasioderma serricorne* and 28.53 mg/cm^2^ against *Liposcelis bostrychophila*	(Guo et al. 2017)
*Piper baccans* (Piperaceae) essential oil	Juniper camphor acetate (30.71%)	Larvicidal activity against *Aedes aegypti* (Culicidae) larval stage (LC_50_ of 10.68 µg/mL and LC_90_ of 22.11 µg/mL) and AChE inhibitory activity (IC_50_ of 38.37 µg/mL)	(Souza et al. 2024)
*Pulicaria somalensis* aerial parts	Juniper camphor (24.7%)	The IC_50_ values for root growth were 0.6 mg/mL for both *B. pilosa* and *D. aegyptium*. For shoot growth, the values were 1.0 mg/mL for *B. pilosa* and 0.7 mg/mL for *D. aegyptium*	(Assaeed et al. 2020)

### Neuroactive potential

Bioinformatics and docking studies demonstrated that juniper camphor obtained from the berries and leaves of *Myrtus communis* can penetrate the blood–brain barrier and interact effectively with acetylcholinesterase (AChE) and butyrylcholinesterase (BChE). This suggests potential neuroprotective or insecticidal effects due to the inhibition of cholinesterase activity (Hussein et al. 2023). This was relevant for both therapeutic and agricultural applications. The essential oil extracted from the capitulum of *Chrysanthemum morifolium*, which was grown in Tongxiang City, contained common chemical components such as juniper camphor (Guo et al. 2008). Additionally, it demonstrated strong neuroprotective properties, making it a potential candidate for the treatment of neurodegenerative conditions such as Parkinson’s disease (Lawal et al. 2014). The steam distillate derived from the flowers of *Dipterocarpus cornutus* Dyer was obtained through steam distillation of 250 g of air-dried flowers, resulting in isolation of juniper camphor (Bakhtiar et al. 2004). The anticholinesterase and anti-inflammatory properties were assessed using the Ellman method and the lipoxygenase (LOX) enzyme, respectively. The essential oil displayed weak inhibitory effects on acetylcholinesterase (AChE) (I%: 30.2%) and butyrylcholinesterase (BChE) (I%: 32.5%), while it exhibited moderate inhibition for LOX (I%: 70.2%). This study presents a novel methodology and results that may assist in the characterization, pharmaceutical, and therapeutic uses of the essential oil from the *Dipterocarpus* genus (Wmnhw et al. 2020).

### Anticancer power

The fractionated extract from *Conamomum vietnamese* rhizome yielded juniper camphor at a concentration of 3.11% as determined by gas chromatography–mass spectrometry analysis, alongside colorimetric and precipitation reactions. This extract, which was fractionated utilizing *n*-hexane, exhibited cytotoxic effects on five distinct human cancer cell lines. These findings indicated that the rhizomes of *C. vietnamense* and their principal components may represent a promising avenue for cancer therapy (Chen et al. 2025). There is no many direct study on juniper camphor and its anticancer effects in the provided literature. However, various similar eudesmane-type sesquiterpenoids have been examined for cytotoxic and anticancer effects, and these findings can help infer potential processes and relevance. Related eudesmane sesquiterpenes exhibit selective anticancer effects. For example, in *Dysoxylum gaudichaudianum*, certain compounds have shown activity against human HeLa cervical cancer, as demonstrated using the Resazurin-based PrestoBlue assay. When compared to cisplatin as a positive control, these compounds displayed moderate cytotoxicity, with an IC_50_ value of 28.04 µM, while another exhibited comparatively weaker activity, with an IC_50_ value of 58.37 µM. A structure–activity relationship analysis indicates that hydroxylation at the C-6 position enhances cytotoxic activity, whereas the presence of an olefinic moiety between C-6 and C-7 reduces potency, likely due to increased molecular rigidity. This highlights the key structural features that modulate activity within the eudesmane scaffold (Maira et al. 2025). An eudesmane-type sesquiterpene lactone isolated from *Inula britannica* shows cytotoxic activity against diffuse large B cell lymphoma cells *in-vitro*. It increases mitochondrial membrane potential depolarization and upregulates intracellular reactive oxygen species (ROS), inhibiting cell growth in OCI-LY3 lymphoma cells by blocking NF-κB signaling. The compound induces apoptosis by activating BAX and cleaved caspase-3 while reducing BCL-2, BCL-XL, and procaspase-3 levels. These findings suggest its potential as an anticancer treatment for lymphoma through ROS-dependent apoptosis and SubG0/G1 arrest in OCI-LY3 cells (Jang et al. 2020). The cytotoxic potential of new eudesmane sesquiterpenes obtained from the Soft Coral *Nephthea* sp. demonstrated moderate cytotoxicity against the MCF-7 and HT-29 cell lines, presenting IC_50_ values of 51.5 ± 2.1 µM and 64.1 ± 1.9 µM, respectively. It showed limited activity against HepG2 cells, with an IC_50_ value of 71.3 ± 2.5 µM (Eissa et al. 2026).

## Pharmaceutical relevance beyond chemical interest

Juniper camphor is classified as an eudesmane sesquiterpene, a type of terpenoid compound known for its significant biological activities. Many sesquiterpenes have demonstrated anti-inflammatory, antimicrobial, and anticancer properties, highlighting their importance in pharmaceuticals. From a medicinal chemistry perspective, the unique structural features of sesquiterpenes such as their diverse carbon skeletons and functional groups are crucial in determining their biological activity. Key advantages of these compounds include targeted cytotoxicity, with mechanisms identified as iNOS inhibition, NF-κB regulation, and membrane disruption. These aspects facilitate informed drug optimization, support a long history of use in traditional medicine, and provide established methods for synthesis. Juniper camphor specifically addresses critical issues related to triple-negative breast cancer, antibiotic resistance, neuroinflammation, and chronic inflammation, making it a promising candidate for organized pharmaceutical development (Aleksandrovich et al., 2021; Kim et al., 2013; Min et al., 2025; Qi et al., 2022).

## Limitations and future directions

This review highlights several important gaps in the current body of literature concerning juniper camphor. Most of the available evidence is derived from previously published studies, with limited reports involving the isolation of highly purified juniper camphor under controlled experimental conditions. The absence of standardized isolation procedures in the literature restricts the ability to assess issues related to compound purity, stereochemistry, and reproducibility across studies. In addition, there is a scarcity of well-designed *in-vitro* investigations specifically evaluating the biological activities of isolated juniper camphor. Controlled cell-based assays are essential to establish dose response relationships and to clarify potential mechanisms of action. Likewise, limited *in-vivo* data are available to evaluate pharmacokinetic properties, bioavailability, safety profiles, and organ-specific effects. Such studies are crucial to better define the therapeutic potential of this compound. Moreover, mechanistic insights at the molecular level remain insufficient, and current evidence does not comprehensively identify the relevant targets and signaling pathways involved. Future research should prioritize the isolation of chemically characterized juniper camphor, followed by systematic *in-vitro* and *in-vivo* investigations to address these knowledge gaps and provide more robust evidence.

## Conclusions

Juniper camphor (eudesm-7(11)-en-4-ol) is a sesquiterpene alcohol with a range of reported biological activities, including antimicrobial and anti-inflammatory effects. Critical gaps include a lack of standardized isolation protocols, insufficient *in-vitro* assays to establish dose response relationships, and scarce *in-vivo* studies to evaluate pharmacokinetics, safety, and organ-specific effects. Mechanistic insights at the molecular level are also minimal, limiting a comprehensive understanding of its biological actions.

Future research should focus on isolating chemically characterized juniper camphor from verified sources, conducting systematic *in-vitro* and *in-vivo* investigations, and elucidating molecular targets and pathways. Addressing these gaps will clarify its therapeutic potential and support the development of natural product-based applications. By providing precise experimental evidence, future studies could establish juniper camphor as a viable candidate for medical and industrial use.

## Data Availability

Data sharing is not applicable to this article as no new data were created or analyzed in this study.
